# Isolation, biological and whole genome characteristics of a Proteus mirabilis bacteriophage strain

**DOI:** 10.1186/s12866-023-02960-4

**Published:** 2023-08-08

**Authors:** Xirui Hao, Xin Cen, Min He, Yongping Wen, Huanrong Zhang

**Affiliations:** 1 College of Animal and Veterinary Sciences, Southwest Minzu University, Chengdu, 610041 Sichuan China; 2 Key Laboratory of Veterinary Medicine of Universities in Sichuan, Chengdu, PR China; 3 College of Food and Biological Engineering, Chengdu University, Chengdu, 610106 Sichuan China

**Keywords:** *Proteus mirabilis* phage, Isolation and identification, Biological characteristics, Whole genome analysis

## Abstract

*Proteus mirabilis*, a naturally resistant zoonotic bacterium belonging to the *Enterobacteriaceae* family, has exhibited an alarming increase in drug resistance. Consequently, there is an urgent need to explore alternative antimicrobial agents. Bacteriophages, viruses that selectively target bacteria, are abundant in the natural environment and have demonstrated potential as a promising alternative to antibiotics. In this study, we successfully isolated four strains of *Proteus mirabilis* phages from sewage obtained from a chicken farm in Sichuan, China. Subsequently, we characterized one of the most potent lytic phages, Q29, by examining its biological and genomic features. Comparative genomic analysis revealed the functional genes and phylogenetic evolution of Q29 phages. Our findings revealed that *Proteus mirabilis* bacteriophage Q29 possesses an icosahedral symmetrical head with a diameter of 95 nm and a tail length of 240 nm. Moreover, phage Q29 exhibited stability within a temperature range of 37 ℃ to 55 ℃ and under pH conditions ranging from 4 to 9. The optimal multiplicity of infection (MOI) for this phage was determined to be 0.001. Furthermore, the one-step growth curve results indicated an incubation period of approximately 15 min, an outbreak period of approximately 35 min, and an average cleavage quantity of approximately 60 plaque-forming units (PFU) per cell. The genome of phage Q29 was found to have a total length of 58,664 base pairs and encoded 335 open reading frames (ORFs) without carrying any antibiotic resistance genes. Additionally, genetic evolutionary analysis classified phage Q29 within the family *Caudalidae* and the genus *Myotail*. This study provides valuable research material for further development of *Proteus mirabilis* bacteriophage biologics as promising alternatives to antibiotics, particularly in light of the growing challenge of antibiotic resistance posed by this bacterium.

## Introduction

*Proteus* is a Gram-negative bacterium belonging to the *Enterobacteriaceae* family. It is characterized by its non-capsulated, spore-free, and pleomorphic nature [1, 2]. Among the *Proteus* species, *Proteus mirabilis* is the most prevalent pathogen and is commonly found in various natural environments such as sewage, feces, and the gastrointestinal tract of humans and animals [1, 2]. In individuals with compromised immune systems, *Proteus mirabilis* can cause a range of diseases, including urinary tract infections, infectious stones, otitis externa, bacteremia, and endocarditis [3–5]. Despite the widespread use of antibiotics for preventing and controlling *Proteus mirabilis* infections, studies have shown that some antibiotics are ineffective against this bacterium due to the presence of biofilms. Moreover, bacteria within biofilms exhibit high levels of resistance to biocides and antimicrobial agents [6, 7]. Consequently, there is a pressing need for novel antimicrobial biologics that are targeted, safe for zoological use, and environmentally friendly to replace antibiotics in the treatment of *Proteus mirabilis* infections [8, 9].

Infections caused by multidrug-resistant bacteria have emerged as a significant global health concern, contributing to a substantial burden of morbidity and mortality. It is estimated that approximately 700,000 deaths occur each year as a result of these infections [10, 11]. Recognizing the gravity of the situation, the World Health Organization (WHO) has identified critical priority pathogens that necessitate the development of new antibiotics or alternative therapeutic options. These pathogens, which include multidrug-resistant bacteria, pose a particular threat in healthcare settings such as hospitals, nursing homes, and for patients reliant on ventilators and intravenous catheters [12]. The Organization for Economic Cooperation and Development (OECD) has projected that if current rates of bacterial resistance persist, 2.4 million people in Europe, North America, and Australia could die between now and 2050 [13]. This alarming statistic highlights the urgent and global nature of the bacterial resistance problem [14]. Efforts to combat bacterial resistance are crucial for safeguarding public health worldwide.

Bacteriophages, as potent natural predators of bacterial populations, are generally considered safe compared to conventional antibiotics since they do not infect humans or other animals [15, 16]. Moreover, bacteriophages offer cost-effective solutions with simple storage and preparation methods, making them highly valuable in disease control, livestock production, and the food industry [17]. Bacteriophage therapy has gained significant attention as a novel approach to combat multidrug-resistant bacteria that have developed resistance to traditional antibacterial agents [18, 19]. Recent studies have demonstrated the potential of bacteriophages in treating infections caused by various multidrug-resistant pathogens, including *Klebsiella pneumoniae* [20], *Pseudomonas aeruginosa* [21], *Acinetobacter baumannii* [22], *Staphylococcus aureus* [23], and *Proteus mirabilis* [24]. For instance, Song et al. [24] demonstrated that a bacteriophage cocktail comprising three canine-derived *Proteus mirabilis* bacteriophages effectively targeted and eradicated *Proteus mirabilis* biofilms, displaying strong bactericidal properties. Similarly, Esmael and Gomaa [25–7] reported positive efficacy of bacteriophages in controlling and eliminating *Proteus mirabilis* biofilms. However, the isolation of *Proteus mirabilis* phages from chicken sources remains relatively unexplored. Therefore, the discovery of chicken-origin *Proteus mirabilis* phages and the exploration of their biological characteristics hold significant importance in this field of research.

In this study, we focused on isolating bacteriophages specific to *Proteus mirabilis* from effluent obtained from a chicken farm. Our investigation involved a comprehensive analysis of the biological and genome-wide characteristics of these bacteriophages. The findings from our study hold great potential in serving as a theoretical foundation for the development of targeted antibacterial preparations specifically designed to combat *Proteus mirabilis* infections.

## Materials and methods

### Bacterial strains and growth media

LB semi-solid medium, SM buffer, PEG 8000, 0.22 μm sterile disposable needle filter and viral genome DNA/RNA extraction kit were purchased from Shenggong Bioengineering, Shanghai, China. Twenty-seven strains of multidrug-resistant *Proteus mirabili* were isolated from chicken and preserved in the laboratory for determining the bacteriophage host spectrum.

### Bacteriophage enrichment and purification

To enrich bacteriophages, a 1000 mL sample of sewage obtained from a chicken farm was taken and CaCl2 was added to achieve a final concentration of 1 mmol/L. The solution was subjected to centrifugation at 7000 ×g for 15 min to remove impurities. The resulting centrifuged sewage was then concentrated overnight using a dialysis bag and sterilized through a 0.22 μm filtration membrane. Subsequently, 100 mL of the filtrate was mixed thoroughly with 100 mL of 2×LB liquid culture. Following this, 100 μL of *Proteus mirabilis* in the logarithmic growth stage was added to the mixture, which was then incubated at 160 r/min and 37 °C for 8 h to facilitate bacteriophage enrichment. This enrichment process was repeated three times to ensure the acquisition of a culture medium containing bacteriophages.

The bacteriophages were separated and purified using the double-layer agar plate method, as described by reference [26]. Initially, 200 μL of the filtrate was thoroughly mixed with 100 μL of host bacteria and incubated at room temperature for 5 min. The mixture was then introduced into 6 mL of LB semi-solid medium at 50 °C, stirred well, and swiftly poured onto LB solid medium to form a double-layer agar plate upon solidification. The double-layer agar plate was inverted and placed in a constant incubator set at 37℃ for 8 to 10 h to allow plaque formation. A single plaque exhibiting a regular shape, translucent appearance, and well-defined edges was carefully selected and soaked in 500 μL of SM buffer. This mixture was incubated overnight at 4 °C. Subsequently, 100 μL of the culture was extracted and used to purify the bacteriophage using the bilayer agar plate method. Through a process of repeated separation and purification, the bacteriophages with higher purity were obtained after undergoing more than six rounds of purification.

### Phage titer determination

The purified bacteriophage lysate was subjected to a 10-fold dilution series. Each dilution was mixed with a separate host bacterial solution and incubated on double-layer agar plates for 8 to 10 days. Plates with a plaque count ranging from 30 to 300 were selected for the enumeration of phage titers. The phage titers were calculated using the following formula: Phage titer (PFU/mL) = number of plaques × dilution factor × 10. This trial was repeated three times to ensure accuracy and reliability of the results.

### Transmission electron microscopy observation

For electron microscopy analysis, a 10 μL suspension of the phage (approximately 10^9 PFU/mL) was applied onto a copper mesh and allowed to naturally precipitate for 15 min. Subsequently, the sample was stained with a 2% phosphotungstic acid solution for 5 min. Excess liquid was carefully removed by blotting with filter paper. Following the drying process, the sample was observed under a transmission electron microscope, and images were captured to record the morphological features of the bacteriophages.

### Stability studies

For the temperature stability test, 100 μL of phage lysate (approximately 10^9 PFU/mL) was incubated in a water bath at different temperatures: 37 °C, 45 °C, 55 °C, 65 °C, 75 °C, and 85 °C. Incubation durations were either 30 or 60 min. After the incubation period, the lysate was diluted 10-fold, and the phage titer change was assessed to evaluate the thermal stability of the bacteriophages.

For the pH stability test, 100 μL of phage lysate (approximately 10^9 PFU/mL) was added to 900 μL of LB liquid medium adjusted to different pH levels ranging from 1 to 12. The mixture was then incubated in a constant temperature water bath at 37 °C for 1 h before sampling. Following incubation, the pH was readjusted to match the original medium pH using HCl (1 mol/L) and NaOH (1 mol/L). The sample was subsequently diluted 10-fold, and the phage titer changes were detected to evaluate the phage’s acid-base tolerance. The test was repeated three times to ensure reliability and consistency of the results.

### Optimal multiplicity of infection (MOI) detection and one-step growth curve determination

The bacteria were cultured until they reached the logarithmic growth stage. Subsequently, the bacteriophage and bacterial solutions were mixed in different proportions: 0.0001, 0.001, 0.01, 0.1, 1, 10, 100, and 1,000. The resulting mixtures were cultured in 5 mL of LB liquid medium for 6 h at 160 r/min and 37 °C. After incubation, the cultures were filtered using a 0.22 μm sterilization filter to obtain the phage lysate. The number of plaques formed was determined using the double-layer agar plate method, and the proportion that resulted in the highest phage titer was identified as the optimal MOI (Multiplicity of Infection). This experiment was repeated three times to ensure reliability and consistency of the results.

The bacterial solution was mixed with the optimal MOI bacteriophage lysate, followed by centrifugation at 7000 × g for 5 min. The supernatant was then discarded, and the resulting pellet was resuspended in 5 mL of LB liquid medium. The suspension was incubated at 37 °C with constant shaking at 160 r/min on a shaker. To generate a one-step growth curve, phage titers were sampled at different time points ranging from 0 to 100 min, with sampling occurring every 5 min. Each time point was repeated three times to obtain an average value. The resulting data were plotted, with the infection time represented on the x-axis and the phage titer on the y-axis. From the one-step growth curve, the latent period, burst size, and lysis rate of the phage can be determined. The latent period refers to the time between phage infection and the onset of phage release, while the burst size represents the average number of phages released per infected bacterium. The lysis rate can be calculated by dividing the phage titer at the end of the outbreak by the initial host bacterial concentration.

### Phage lysis spectrum assay

The cleavage ability of *Proteus mirabilis* phages against 27 strains of *Proteus mirabilis* was determined by double-layer agar plate method.

### Analysis of phage genomes

4 mL of bacterial solution and 10 mL of phage lysate (109 CFU/mL) were combined with 400 mL of LB liquid medium, according to the optimal MOI. The mixture was incubated at 37 °C with shaking at 180 r/min for 18 h. Following incubation, the culture medium was centrifuged at 8000 r/min at 4 °C for 20 min, and the resulting supernatant was collected. To the sedimentation, 22.23 g of NaCl was added to achieve a final concentration of 1 mol/L. The bacterial fragments were then precipitated by placing the mixture in an ice bath for 1 h after complete dissolution. Subsequently, the culture was centrifuged again at 8000 r/min at 4 °C for 20 min, and the supernatant was transferred to a sterilized Erlenmeyer flask. A total of 37 g of PEG 8000 was added at a ratio of 10% (w/v) and fully dissolved. The mixture was then immersed in an ice bath for 3 h to concentrate the phages. The culture was subsequently centrifuged at 10,000 × g at 4 °C for 10 min, and the resulting pellet was dissolved in 2 mL of SM buffer. Following filtration through a 0.22 μm filter, 2 mL of chloroform was added at a ratio of 1:1. The mixture was gently shaken for 1 min to allow chloroform to function effectively. It was then subjected to centrifugation at 955 × g for 15 min. This step was repeated 2–3 times to ensure complete removal of proteins from the phage. Finally, the hydrophilic phase was concentrated for use in the extraction of the bacteriophage genome.

The bacteriophage genome was extracted using a viral genome DNA/RNA extraction kit. Its purity was assessed through 1% agarose gel electrophoresis, ensuring a genomic quantity of no less than 2 μg for sequencing. The extracted genomic DNA was then sent to Beijing Nuohe Zhiyuan Biotechnology Co., Ltd. for whole genome sequencing. Subsequently, the phage genome sequence was assembled to obtain the complete genome sequence, which was uploaded to NCBI (https://www.ncbi.nlm.nih.gov/). Various bioinformatics tools were employed for genome analysis. Prodigal [27] was used to predict coding sequences (CDS), Signal [28] was utilized for signal prediction, and infernal [29] and RNAmmer [30] were employed for predicting tRNA, rRNA, and ncRNA. The whole genome sequence of bacteriophage Q29 was analyzed using EditSeq [31], which included the analysis of base composition, GC content, and the prediction of tRNA using tRNAscan-SE [32]. The open reading frames (ORFs) of the bacteriophages were predicted using ORF finder (https://www.ncbi.nlm.nih.gov/orffinder/). The predicted ORFs were then verified and annotated using Smart Blast. SnapGene was employed for comprehensive annotation and visual analysis of the whole genome to understand the gene function and distribution characteristics at a macroscopic level. To determine the evolutionary relationship, the genome sequence of the large subunit gene of the terminal enzyme in the conserved region of the phage whole genome sequence was selected and compared using NCBI. The evolutionary tree was constructed using MEGA7.0 software, specifically utilizing the Neighbor-Joining method with 500 Bootstrap tests to assess reliability. The top 10 similarities were considered for the analysis.

### Statistical analysis

The statistical analysis results were presented as means ± standard deviation (SD). Differences between the two groups were assessed using an unpaired T-test. The statistical analysis was performed using Graphics 8.0 software, and a p-value less than 0.05 (P < 0.05) was considered statistically significant.

## Results

### Phage isolation and purification

**Fig. 1:**
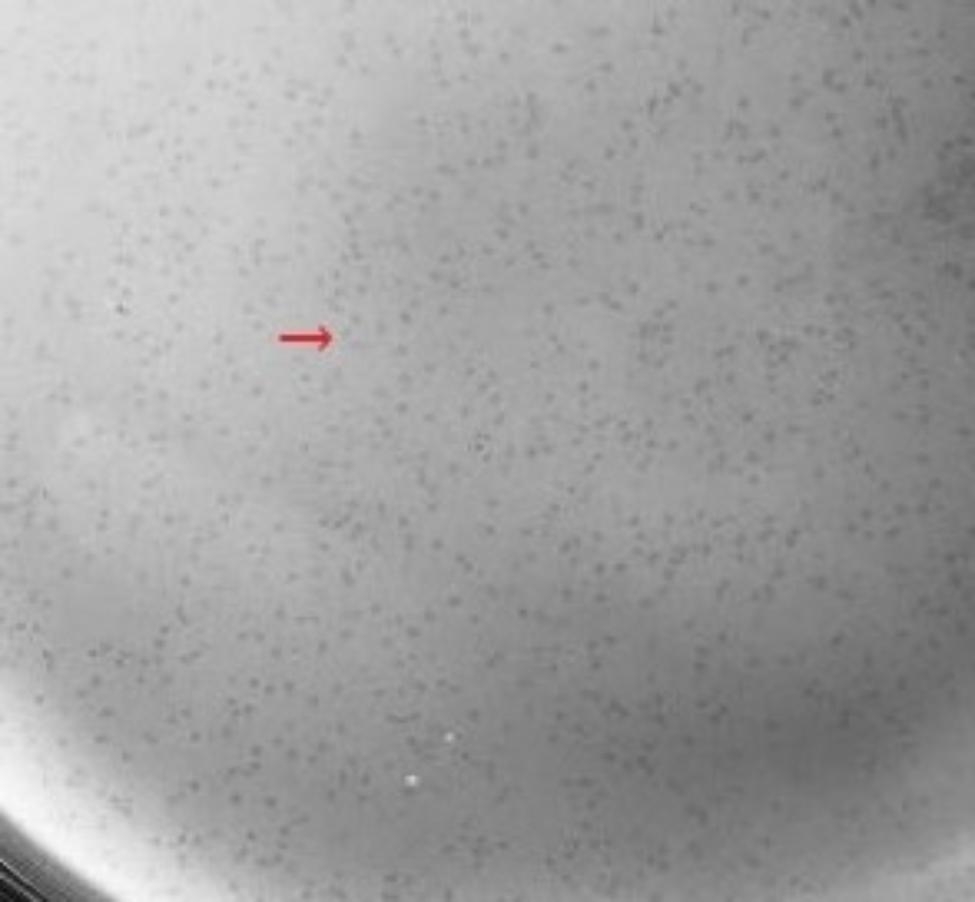
Plaques formed by bacteriophages of *Proteus mirabilis* Each dot represented a transparent plaque with regular shape, neat edges, and similar size on double-layer agar plate after 6 rounds of phage purification. The red arrow indicated Q29 plaque morphology.

*Proteus mirabilis*, specifically chosen as the host bacteria, was utilized to isolate bacteriophages from chicken farm sewage. Following six rounds of phage purification, distinct plaques with consistent features, including regular shape, well-defined edges, and similar sizes, were observed on double-layer agar plates. Four strains of *Proteus mirabilis* phages were successfully isolated and obtained. Notably, one of the phages exhibited a plaque diameter of approximately 1.2 mm (Fig. 1) and was designated as Q29.

### Phage Q29 morphology and species

**Fig. 2:**
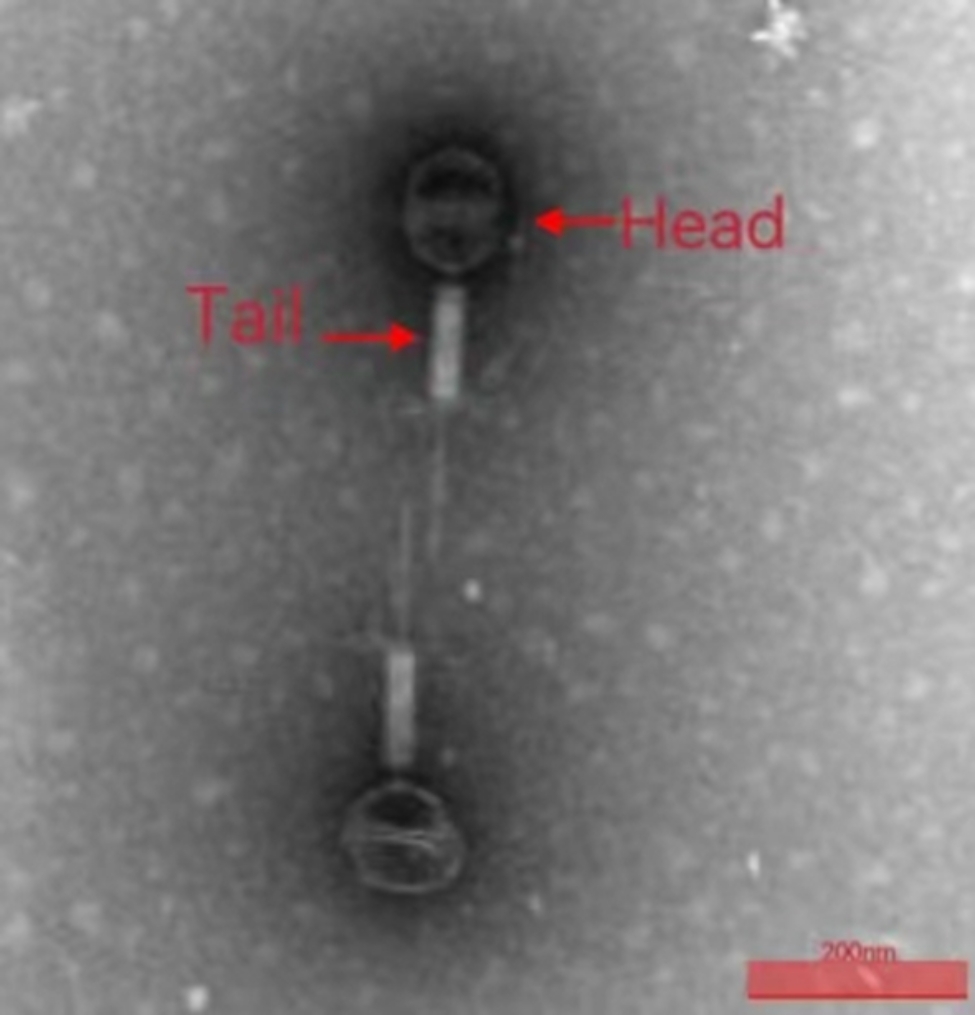
Electron micrograph of *Proteus mirabilis* phage Q29 The bacteriophage particle (indicated arrows) exhibited a head of icosahedral symmetry with a diameter of 95 nm and a tail length of 240 nm. Scale bar = 200 nm.

Morphological analysis using Transmission Electron Microscopy (TEM) demonstrated that bacteriophage Q29 exhibited an orthoicosahedral symmetrical head with a diameter of 95 nm and a tail measuring 240 nm in length (Fig. 2). Based on the classification guidelines outlined by the International Committee on Taxonomy of Viruses (ICTV), the bacteriophage Q29 was classified within the family *Caudalidae* and the genus *Myoceragodons*.

### Phage Q29 titer

The phage Q29 lysate was subjected to a 10-fold dilution, and its titer was measured as 3 × 10^9^ PFU/mL by the double-layer agar plate method.

### Phage Q29 lysis spectrum

To further investigate the biological characteristics of phage Q29, we conducted a lysis spectrum analysis. The results revealed that bacteriophage Q29 was able to successfully lyse 12 out of the 27 tested multidrug-resistant *Proteus mirabilis* strains, indicating a lysis rate of 44% (12/27). The lysis spectrum of phage Q29 was found to be broad. Detailed lysis results can be found in Table 1.

**Table 1:** Lysis ability of phage Q29 on 27 strains of *Proteus mirabilis.*

Bacterial number	1	2	3	4	5	6	7	8	9	10	11	12	13	14	15	16	17	18	19	20	21	22	23	24	25	26	27
**Lysis results**	**+**	**+**	**+**	**-**	**-**	**+**	**+**	**-**	**+**	**+**	**-**	**-**	**+**	**-**	**-**	**-**	**-**	**+**	**+**	**-**	**-**	**-**	**+**	**-**	**-**	**-**	**+**

The 27 strains of *Proteus mirabilis* isolated and preserved in the laboratory were numbered 1 ~ 27. “+”: Lysis successful, “-”: Lysis failed.

### Phage Q29 thermal and pH stability

**Fig. 3:**
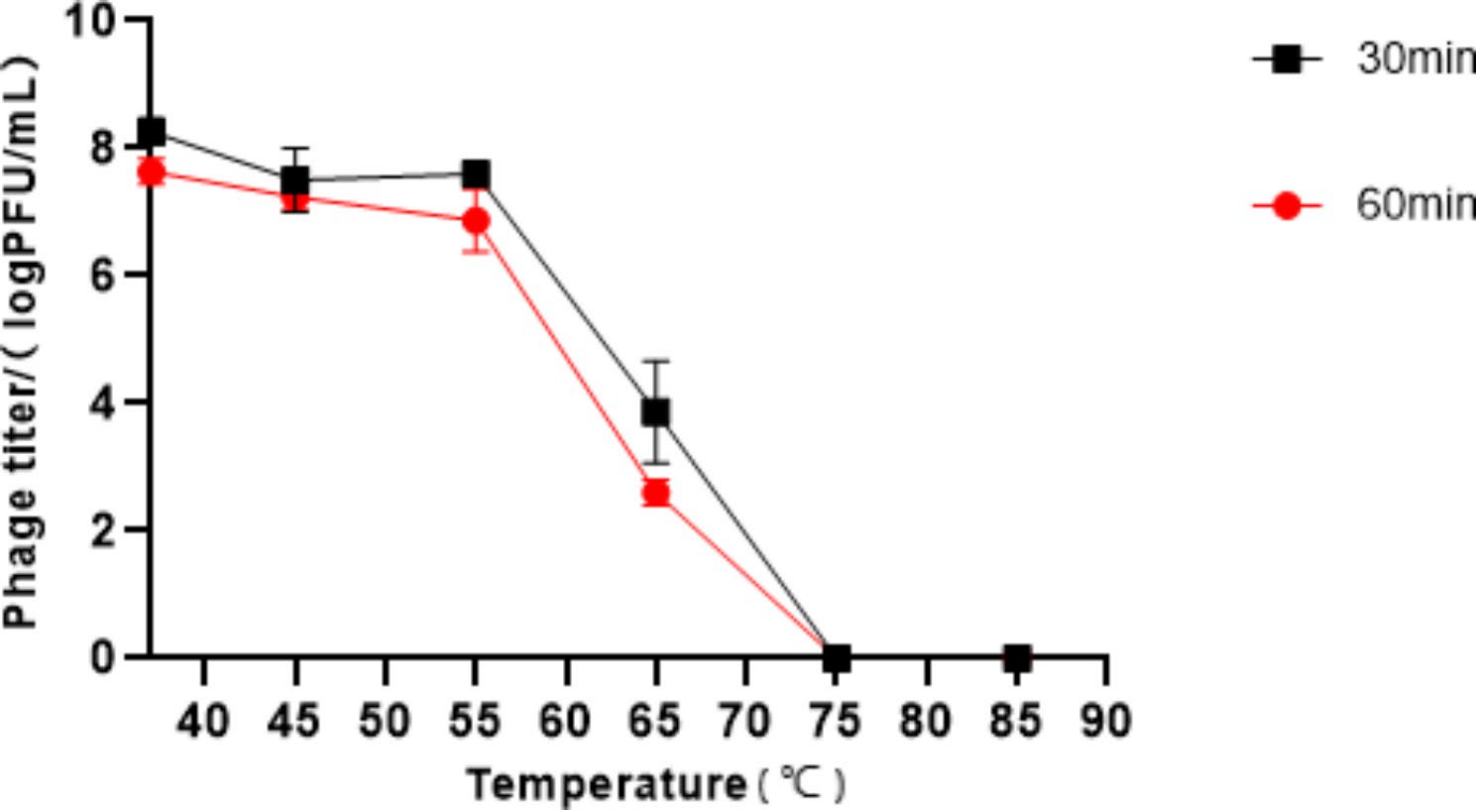
Determination of thermal stability of phage Q29 At different temperatures, phage Q29 acted on the cleavage of host bacteria for 30 and 60 min. Phage lysis time: (■) 30 min, (●) 60 min. Data represented the mean ± SD of three independent experiments was shown.

In this study, we conducted an assessment of the stability of phage Q29 under elevated temperature conditions and varying pH levels. After subjecting the phage to temperatures ranging from 37 to 75 °C for 30 or 60 min, we observed that the activity of bacteriophage Q29 remained relatively stable and exhibited good heat resistance up to 55 °C. However, beyond 55 °C, the phage titer declined rapidly with time, and complete inactivation occurred after exposure to 75 °C. Interestingly, the phage titer did not show a significant reduction even after being incubated for 60 min compared to 30 min (as shown in Fig. 3). These findings suggest that phage Q29 possesses a certain level of thermal stability.

**Fig. 4:**
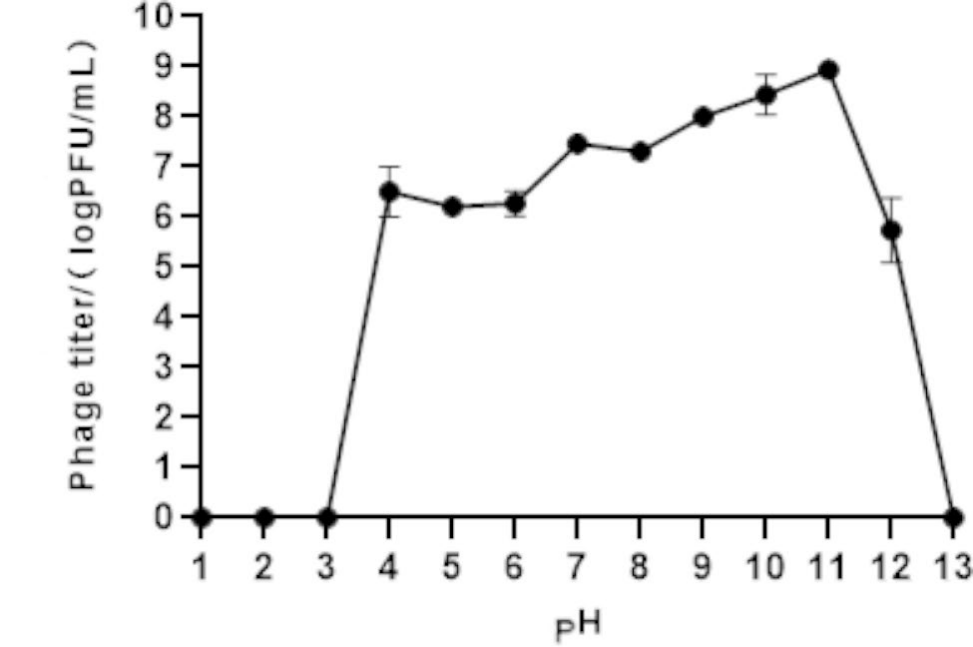
Determination of pH stability of phage Q29 Dynamics of phage Q29-mediated lysis of *Proteus mirabilis* under different pH conditions. Data represented the mean ± SD of three independent experiments was shown.

Furthermore, our investigation revealed that phage Q29 maintained high titers (> 6 log10 PFU/mL) within the pH range of 4 to 11 for up to 1 h. Notably, the highest titer value was observed at pH 11, reaching 8.8 log10 PFU/mL. However, when exposed to pH values of 1 to 2 or 13, the phage activity was completely lost (as depicted in Fig. 4). These findings indicate that phage Q29 exhibits excellent stability at various pH levels and further underscore its remarkable pH and thermal stability.

### The optimal MOI and one-step growth curve of phage Q29

Subsequently, we conducted a series of experiments to determine the optimal Multiplicity of Infection (MOI) by mixing the phage with logarithmic phase *Proteus mirabilis* at various ratios. Our results indicated that the highest phage titer, reaching 5 × 1013 PFU/mL, was achieved at an optimal MOI of 0.001. Hence, the optimal MOI for phage Q29 was determined to be 0.001.

**Fig. 5:**
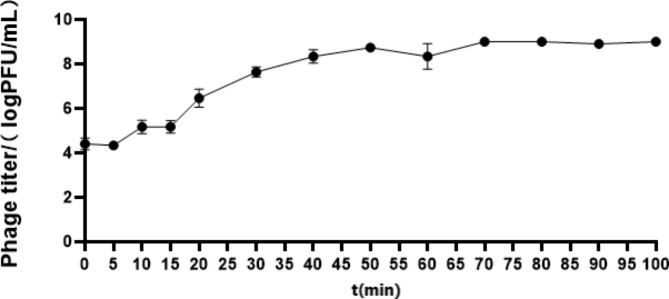
One-step growth curve determination of phage Q29 One-step growth curve was drawn according to the ratio of MOI = 0.001.

To evaluate the infection potential of phage Q29, we conducted a one-step growth curve assay at an MOI of 0.001. The results, depicted in Fig. 5, demonstrated that the phage titer remained stable during the initial 15 min of the incubation period following infection of the host bacteria. Subsequently, between 15 and 50 min of incubation, there was a significant increase in phage proliferation, resulting in a substantial average lysis amount of 60 PFU·cell-1 (lysis amount = phage titer at the end of the outbreak / the initial host bacterial concentration). The outbreak period, characterized by substantial phage replication, was approximately 35 min. After 50 min, the bacteriophage titer remained relatively constant, indicating the entry into a stable phase (Fig. 5). These findings illustrate that phage Q29 possesses a short incubation period and a high lysis rate.

### Whole-genome and functional ORF analysis

**Fig. 6:**
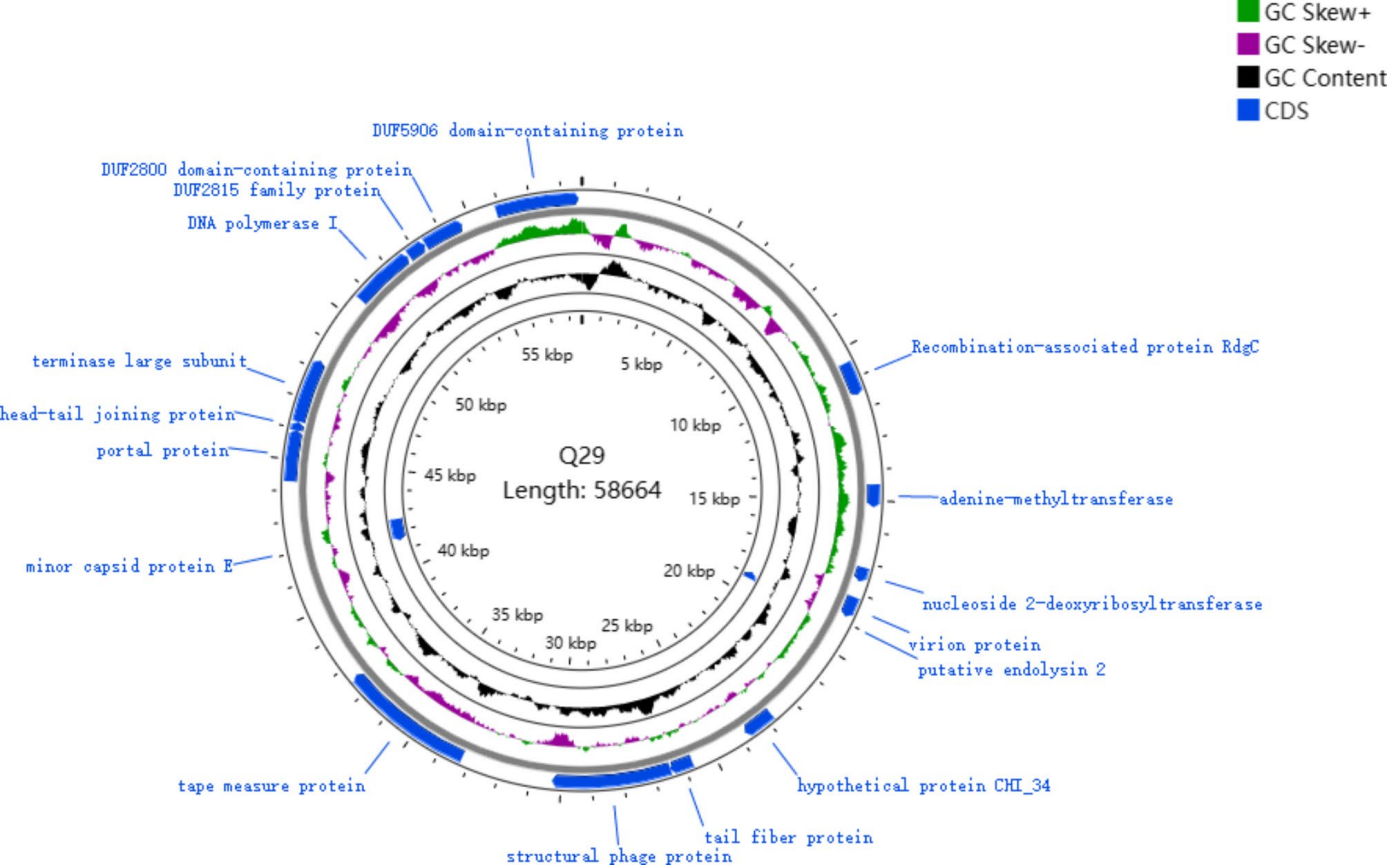
Circular genome map of phage Q29 Circular genome representation of the *Proteus mirabilis* phage Q29. The innermost circle indicated the GC content (black). The second circle indicated GC skew on positive and negative chains (green and purple). The outer ring represented the predicted CDS located on positive and negative DNA strands. Blue indicated tRNA-coding genes.

**Fig. 7:**
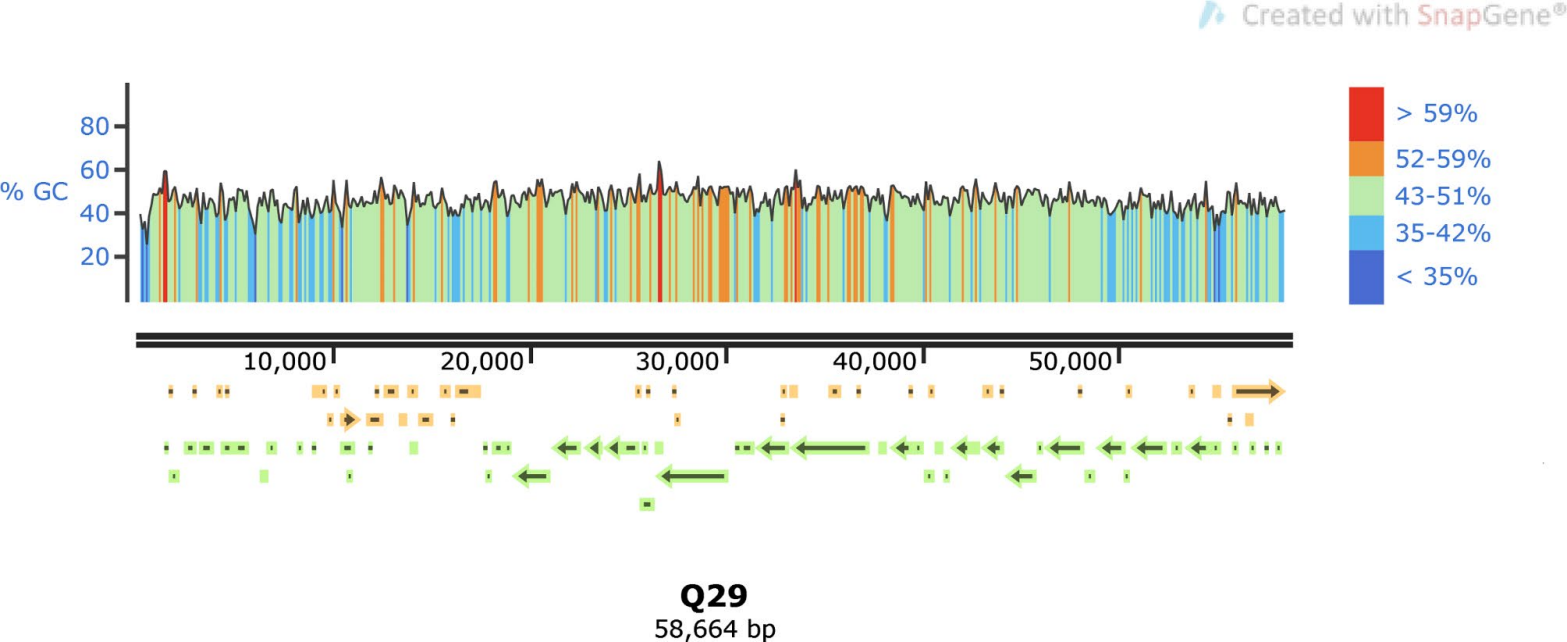
Genome linear diagram of phage Q29 Yellow arrows represented positive directions, green arrows represented negative directions, dark blue indicated GC content < 35%, light blue indicated GC content between 35 and 42%, green indicated GC content between 43 and 51%, orange indicated GC content between 52 and 59%, red indicated GC content > 59%.

Whole-genome sequencing provided a comprehensive understanding of the features of phage Q29. The analysis revealed that the total length of the phage Q29 genome was 58,664 base pairs (bp). The distribution of bases in the genome was as follows: A (25.14%), G (22.30%), T (27.99%), and C (24.57%), resulting in a GC content of 46.87% (as shown in Figs. 6 and 7). Notably, the prediction by tRNAscan-SE indicated the absence of a gene encoding tRNA in the phage genome, suggesting that protein synthesis in this bacteriophage is entirely reliant on the host’s tRNA machinery. The complete nucleotide sequence of the phage genome was submitted to NCBI and assigned the registration number OM962992.

The predicted open reading frames (ORFs) of phage Q29’s genome and its entire proteome were analyzed using NCBI-Blast annotation. As a result, 30 ORFs were successfully annotated with potential functions. The longest ORF had a length of 4,332 base pairs and was predicted to serve as a caudal tape measure protein. Conversely, the shortest ORF spanned 237 base pairs and was predicted to function as a tail assembly protein. The phage genome was found to be encapsulated by an icosahedral capsid, which was connected to a spiral shrink sheath that enveloped the core tube. The hexagonal base plate, located distal to the contraction sheath, acted as an anchor for the tail and short-tailed fibers. These structures played crucial roles in recognizing and adsorbing to the surface of the host bacteria, facilitating successful infection.

The annotated structural components of phage Q29 encompassed a range of proteins, including tail fibrin (ORF37), tail protein (ORF38), tail assembly protein (ORF40), tape measure protein (ORF43), major capsid protein (ORF50), minor capsid protein (ORF193), portal protein (ORF53), head-tail junction protein (ORF54), and more. Additionally, the phage comprised other domain proteins, such as ORF18, ORF30, ORF41, ORF45, ORF56, ORF58, ORF60, ORF61, ORF64, and others. Furthermore, genes associated with lysis function, including endolysin (ORF262), as well as DNA packaging functions, such as the terminating enzyme large subunit (ORF239), were identified. Phage Q29 also contained pseudoprotein modules, including ORF14, ORF28, ORF34, ORF35, ORF42, ORF46, ORF47, ORF48, and various proteins involved in nucleic acid metabolism and replication, such as N-6-adenine methyltransferase (ORF23), S49 family peptidase (ORF52), helicase (ORF57), and DNA polymerase (ORF59). Notably, no significant similarity was observed to any known antibiotic resistance genes or virulence genes within the phage Q29 genome.

### Phylogenetic analysis

**Fig. 8:**
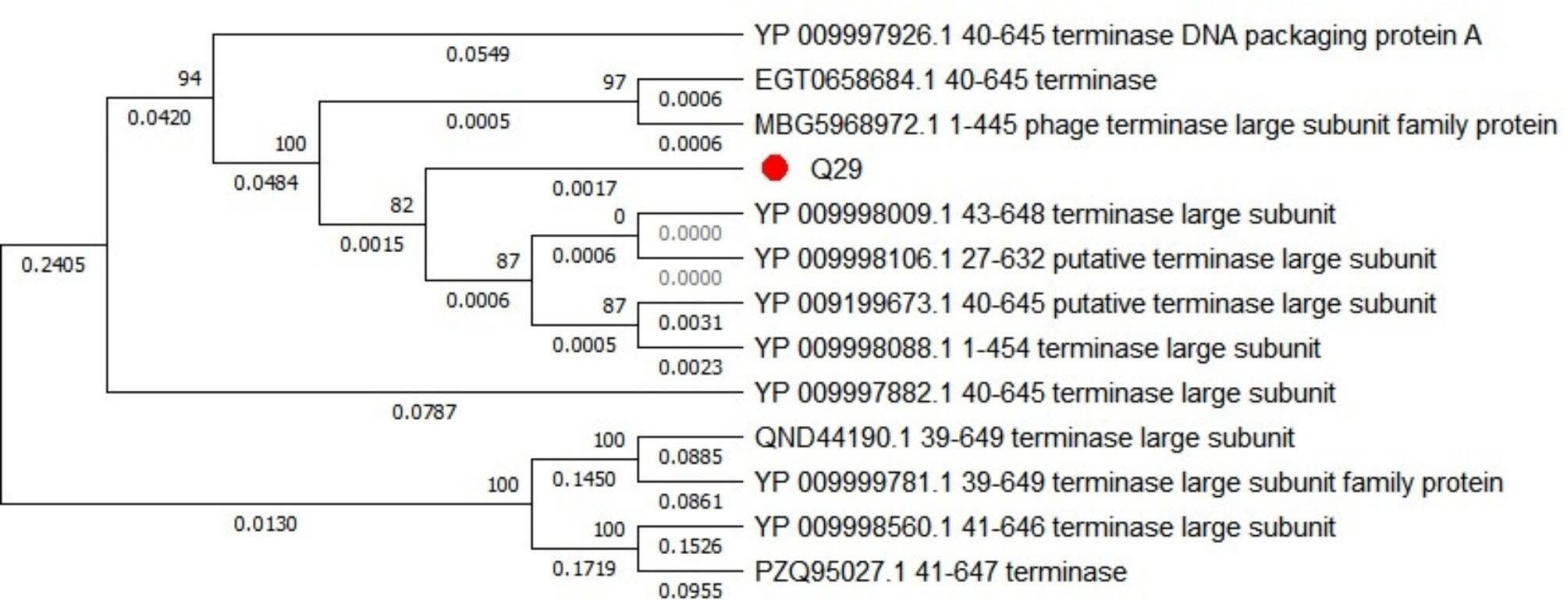
Phylogenetic tree of phage Q29 The gene sequences of the terminal enzyme large subunits were selected from NCBI for comparison. The evolutionary tree was generated using MEGA7.0 software, and the evolutionary tree was constructed via the Neighbor-Joining method for the gene sequences of the top 10 most similar terminal enzyme large subunits. The Bootstrap test was performed 500 times, and the bootstrap value were displayed for each branch of the tree.

The terminal enzyme large subunit plays a crucial role in the DNA packaging machinery of phages and is often conserved among phage genomes, making it suitable for phylogenetic analysis. In our study, we selected the gene sequence encoding the terminal enzyme large subunit from phage Q29 and identified other bacteriophages with high similarity to construct a phylogenetic tree. As depicted in Fig. 8, phage Q29 exhibited high homology with other phages registered in the NCBI database, including YP 009998009.1 43–648, YP 009998106.1 27–632, YP 009199673.1 40–645, and YP 009998088.1 1-454. This observation indicates that bacteriophage Q29 belongs to the family *Caudalidae* and the genus *Myoceragodons*.

## Discussion

*Proteus mirabilis* bacteriophages have shown great promise as an alternative to antibiotics in combating antibiotic-resistant biofilms. These biofilms, which are highly resistant to conventional antibiotics, can be effectively targeted and destroyed by phages. Some phages produce polysaccharide depolymerases, enzymes that degrade the protective exopolysaccharides (EPS) surrounding bacterial cells in biofilms. This degradation allows phages to penetrate and target the bacteria embedded within the biofilm matrix. Once inside, the phages can further disrupt the integrity of the biofilm and lyse the bacterial cells [33]. Previous studies have demonstrated the efficacy of phage cocktails composed of three canine-derived *Proteus mirabilis* phages. These phages specifically target the bacterial membrane structure and have been shown to effectively eliminate bacteria within the biofilm matrix [24]. Additionally, other studies by Esmael A [25] and Gomaa S [7] have reported the successful control and eradication of biofilm-associated *Proteus mirabilis* infections using bacteriophages. However, there is limited knowledge about phages isolated from chicken-derived *Proteus mirabilis*. Therefore, it is crucial to identify and characterize phages originating from this source. Further exploration of chicken-derived *Proteus mirabilis* phages will contribute to our understanding of their potential as therapeutic agents and aid in the development of targeted treatments for *Proteus mirabilis* infections.

The separation rate of bacteriophages was found to be highest in sewage samples compared to fecal and padding samples, indicating that sewage provides a more favorable environment for the survival of bacteriophages [34]. In our study, we successfully isolated and characterized a chicken-derived *Proteus mirabilis* phage, Q29, from Sichuan Province, China. The morphological analysis using electron microscopy confirmed that Q29 displayed typical characteristics observed in *Proteus mirabilis* bacteriophages. The ratio of phage lysate to host bacteria is an important factor during the initial isolation of bacteriophages, typically ranging from 1:1 to 2:1 [35–37]. Interestingly, in our study, we achieved optimal phage enrichment with a ratio of 3:1, which may be attributed to the efficient lysis capabilities of the phages and host bacteria. The titer of Q29 phage reached a high value of 3 × 109 PFU/mL, surpassing the reported values by Li S et al. [38] and Song J et al. [39]. Furthermore, Q29 demonstrated superior thermal stability and acid-base tolerance compared to previous studies. The ability of Q29 to withstand high temperatures and resist gastric acid makes it a promising candidate for the development of bacteriophage biologics [40, 41]. A lower multiplicity of infection (MOI) is preferred to reduce production and application costs of bacteriophages. In our study, we determined the optimal MOI of Q29 to be 0.001, which is consistent with the optimal MOI reported by Lin Z [42]. The one-step growth curve analysis revealed that the outbreak period of phage Q29 was approximately 35 min, similar to the outbreak time observed for the vB_PmM_S strain of *Proteus mirabilis* isolated by Song J et al. [39]. However, Q29 exhibited a shorter incubation period and a greater amount of lysis compared to vB_PmM_S. Overall, the biological characteristics of Q29 surpassed those observed in the *Proteus mirabilis* bacteriophage isolated by Zhou X [43]. These findings highlight the unique and advantageous properties of Q29 as a potential therapeutic agent against *Proteus mirabilis* infections.

Phage genome-wide analysis provides valuable insights into phage protein genes, allowing us to explore the genetic diversity of phage populations, investigate their origins, and understand the evolutionary mechanisms that shape these populations [44]. A recent study conducted by Wasfi R et al. [45] demonstrated the potential of genome-wide analysis in *Proteus mirabilis* bacteriophages, revealing that these phages can enhance infection through the acquisition of receptors or nucleic acid translocations. Furthermore, Corban et al. [46] identified three lysis proteins in the bacteriophage Privateer, including inner- and outer-spanins and an endolysin endopeptidase. Genomic studies have consistently highlighted the ubiquity of endolysin as a lytic protein in *Proteus mirabilis* bacteriophages [47]. In the study by Hatfull [44], it was revealed that bacteria have developed various anti-phage systems to combat phage infection, and the addition of antimicrobial resistance gene elements to phage genomes has been explored as a strategy to address bacterial resistance to bacteriophages. In our research, we conducted whole genome sequencing and sequence analysis of phage Q29. Interestingly, we discovered that the capsid protein, which serves as the main structural protein of the bacteriophage, can be modified with fluorophores, nanoparticles [48], antigens [49], or drugs [50]. Additionally, the large subunit of the terminal enzyme in Q29 exhibited ATPase, endonuclease, and DNA helicase activities, which play critical roles in phage assembly. However, research on phage terminal enzymes remains limited [51], and further investigations are needed to understand the functions of conserved motifs in these enzymes. Overall, the genome-wide analysis of phage Q29 has provided valuable information about its genetic composition and potential applications. It has shed light on the diverse functions of phage proteins and highlighted the need for further exploration in understanding the mechanisms underlying phage-host interactions and the role of conserved motifs in phage biology.

Bacteriophage Q29 exhibits several notable characteristics, including a large lysis capacity, strong lysis ability, and a wide tolerance range to temperature and pH. These features indicate the significant potential of bacteriophage Q29 for the development of bacteriophage biologics targeting *Proteus mirabilis*. The genome-wide analysis of Q29 has also provided insights into the presence of functional protein coding sequences, such as endolysin and large subunits of terminal enzymes. These findings lay the foundation for further research on the functional regulation of viral genes. Importantly, *Proteus mirabilis* bacteriophage Q29 does not carry any drug resistance genes, highlighting its high application safety. This characteristic makes it an excellent candidate for the development of phage preparations, as it circumvents concerns related to antibiotic resistance. The absence of drug resistance genes further supports the potential use of bacteriophage Q29 as an alternative therapeutic option. Overall, the combination of Q29’s robust lysis capabilities, wide tolerance range, absence of drug resistance genes, and promising genomic features positions it as a valuable research target for the development of phage-based interventions against *Proteus mirabilis* infections. Further studies exploring its therapeutic potential and mechanisms of action are warranted.

In conclusion, this study presents significant findings on the isolation and characterization of a chicken-derived *Proteus mirabilis* phage, Q29. The study highlights the superior biological characteristics of phage Q29 compared to previously reported phages. This research contributes to the growing body of knowledge on bacteriophage biologics and emphasizes the importance of exploring phages from diverse sources. By expanding the repertoire of available bacteriophage biologics, we can enhance their efficacy in combating bacterial infections and address the pressing issue of antibiotic resistance. Further investigations in this field will pave the way for the development of innovative and effective phage-based therapies against *Proteus mirabilis* infections.

## Data Availability

The complete genome sequence of *Proteus mirabilis* phage Q29 have submitted to GenBank. The phage genome-wide nucleic acid sequence was uploaded to NCBI and acquired the registration number OM962992 (https://www.ncbi.nlm.nih.gov/nuccore/OM962992). The datasets generated and/or analyzed during the current study are available from the corresponding author on reasonable request.
